# Methyl 2-[(carbamoyl­amino)­imino]-2-(3-{1-[(carbamoyl­amino)­imino]-2-meth­oxy-2-oxoeth­yl}phen­yl)acetate ethanol monosolvate monohydrate

**DOI:** 10.1107/S1600536812001237

**Published:** 2012-01-21

**Authors:** Dilmurot Ismatov, Umarkhon Azizov, Jamshid Ashurov, Samat Talipov

**Affiliations:** a Tashkent Chemical Technology Institute, A. Navoyi 11, Tashkent, Uzbekistan; b Uzbekistan Scientific Research Pharmacological Chemistry Institute (named after A. Sultonov), Durmon Yuli str. 40, Tashkent, Uzbekistan; c Institute of Bioorganic Chemistry, Academy of Sciences of Uzbekistan, M. Ulugbek Street 83, Tashkent, 100125 Uzbekistan

## Abstract

In the title compound, C_14_H_16_N_6_O_6_·C_2_H_6_O·H_2_O, both substit­uents of the benzene ring are approximately planar with maximum deviations from the mean plane of 0.0561 (12) (an imine N atom) and 0.1419 (11) Å (a meth­oxy O atom). The substituents are tilted out of the plane of the benzene ring by 64.48 (4) and 70.08 (5)°, respectively. In the crystal, mol­ecules form centrosymmetric dimers associated *via* pairs of N—H⋯O hydrogen bonds. The dimers are linked *via* the water and ethanol mol­ecules, forming two-dimensional hydrogen-bond networks lying parallel to (100).

## Related literature

For details of the synthesis of 1,4-benzodiketodicarb­oxy­lic acid and its derivatives, see: Ismatov *et al.* (1991 ▶). For the synthesis and biological activity of 1,3-benzodiketodicarb­oxy­lic acid and its derivatives, see: Ismatov *et al.* (1998 ▶, 2001 ▶).
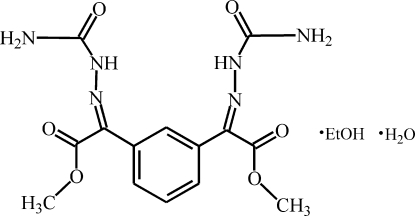


## Experimental

### 

#### Crystal data

C_14_H_16_N_6_O_6_·C_2_H_6_O·H_2_O
*M* *_r_* = 428.41
Monoclinic, 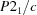

*a* = 7.5810 (2) Å
*b* = 12.1216 (3) Å
*c* = 23.0379 (7) Å
β = 97.254 (3)°
*V* = 2100.11 (10) Å^3^
*Z* = 4
Cu *K*α radiation
μ = 0.94 mm^−1^
*T* = 294 K
0.41 × 0.32 × 0.26 mm

#### Data collection

Oxford Diffraction Xcalibur diffractometer
Absorption correction: multi-scan (*ABSPACK* in *CrysAlis PRO*; Oxford Diffraction, 2009 ▶) *T*_min_ = 0.700, *T*_max_ = 1.000
15863 measured reflections
4317 independent reflections
2927 reflections with *I* > 2σ(*I*)
*R*_int_ = 0.035

#### Refinement

*R*[*F* ^2^ > 2σ(*F* ^2^)] = 0.043
*wR*(*F* ^2^) = 0.125
*S* = 0.98
4317 reflections
298 parameters
H atoms treated by a mixture of independent and constrained refinement
Δρ_max_ = 0.27 e Å^−3^
Δρ_min_ = −0.18 e Å^−3^

### 

Data collection: *CrysAlis PRO* (Oxford Diffraction, 2009 ▶); cell refinement: *CrysAlis PRO*; data reduction: *CrysAlis PRO*; program(s) used to solve structure: *SHELXS97* (Sheldrick, 2008 ▶); program(s) used to refine structure: *SHELXL97* (Sheldrick, 2008 ▶); molecular graphics: *XP* in *SHELXTL* (Sheldrick, 2008 ▶); software used to prepare material for publication: *SHELXL97*.

## Supplementary Material

Crystal structure: contains datablock(s) I, global. DOI: 10.1107/S1600536812001237/fy2032sup1.cif

Structure factors: contains datablock(s) I. DOI: 10.1107/S1600536812001237/fy2032Isup2.hkl

Supplementary material file. DOI: 10.1107/S1600536812001237/fy2032Isup3.cml

Additional supplementary materials:  crystallographic information; 3D view; checkCIF report

## Figures and Tables

**Table 1:** Hydrogen-bond geometry (Å, °)

*D*—H⋯*A*	*D*—H	H⋯*A*	*D*⋯*A*	*D*—H⋯*A*
O1*W*—H1*WA*⋯O6	0.85	2.05	2.879 (3)	166
O1*W*—H1*WB*⋯O5^i^	0.85	2.16	2.941 (3)	152
N3—H3*A*⋯O7	0.86 (4)	2.13 (4)	2.980 (2)	169 (3)
N3—H3*B*⋯O5^ii^	0.85 (3)	2.61 (2)	3.082 (2)	116.7 (18)
N5—H5*A*⋯O3^iii^	0.86	2.19	3.010 (2)	160
N6—H6*A*⋯O1*W*^iv^	0.84 (3)	2.34 (3)	3.137 (3)	158 (3)
N6—H6*B*⋯O7^v^	0.90 (3)	1.99 (3)	2.871 (3)	166 (2)
O7—H7⋯O2	0.88 (3)	1.96 (3)	2.801 (2)	158 (3)
O7—H7⋯N1	0.88 (3)	2.52 (3)	3.0677 (19)	121 (2)
C4—H4⋯O6^vi^	0.93	2.57	3.493 (2)	173
C13—H13*C*⋯N6^vii^	0.96	2.62	3.405 (3)	139

Symmetry codes: (i) 
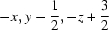
; (ii) 
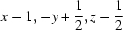
; (iii) 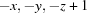; (iv) 
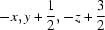
; (v) 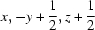; (vi) 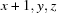; (vii) 
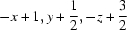
.

**Fig. 1.**
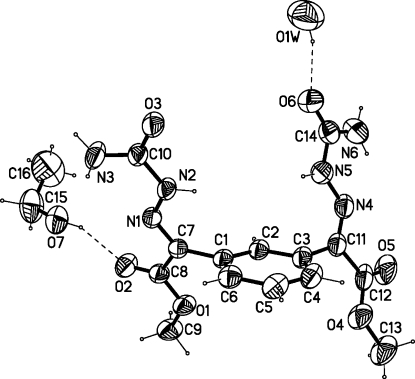
The molecular structure of title compound, with the atom numbering scheme. Displacement ellipsoids are drawn at the 50% probability level.

**Fig. 2.**
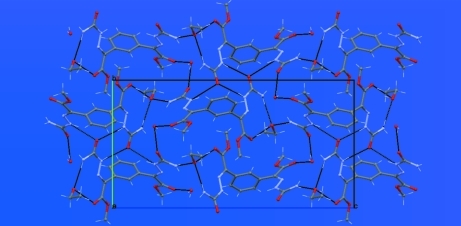
Packing diagram for title compound.

## supplementary crystallographic information

### Comment

It has been reported previously that arylketodicarbonicacid derivatives possess antiinflammatory activity (Ismatov *et al.*, 1991, Ismatov *et al.*, 1998, Ismatov *et al.*, 2001). We report herein the X-ray crystallographic study of a disemicarbazonodimethylether-1,3-benzenediketodicarbonic acid monohydrate ethanol solvate.

The asymmetric unit of the title compound, C_14_H_16_N_6_O_6_.C_2_H_6_O.H_2_O, contains a disemicarbazonodimethylether-1,3-benzenediketodicarbonic acid (DBA), an ethanol and a water molecule as shown in Fig. 1. In the DBA molecule the planes of the substituent fragments (C1/C7/C8/N1/N2/O1/O2 and C3/C11/C12/N4/N5/O4/O5) are tilted out of the mean plane of the benzene ring by 64.48 (4)° and 70.08 (5)°, respectively.

In the crystal structure (Fig. 2), DBA molecules form centrosymmetric pair associates *via* N2—H···O3 and N5—H···O3 H-bonds and the associates are linked via H-bonds by water (O1W—H1···O5, O1W—H2···O6, N6—H···O1W) and ethanol (N6—H···O7, N3—H3···O7,O7—H7···O2) molecules forming two-dimensional networks parallel to the (100) plane (Table 1).

### Experimental

In a round bottom flask, dimethylether-1,3-benzodiketodicarboxylic acid (0.02 mol, 0.51 g) was dissolved in 5 ml methanol. Drops of semicarbazide chloride (0.04 mol, 0.44 g) dissolved in ethanol (5 ml) were then added. The reaction mixture was refluxed for 30 min. The precipitate (0.44 g )formed after 24 h was filtered. The precipitate was then dissolved in ethanol at room temperature. After few days, colorless crystals (m.p. 120–121°C) were formed by slow evaporation.

### Refinement

C-bound H atoms were placed in calculated positions with C—H 0.93 Å for aromatic, 0.97 Å for CH_2_ and 0.96 Å for CH_3_ hydrogens and were refined as riding on their parent atoms, with U_iso_(H) = 1.2 U_eq_(C) or 1.5 U_eq_(C) for methyl H atoms. NH H atoms were placed in calculated positions with N—H 0.86 Å and refined in riding mode with U_iso_(H) = 1.2 U_eq_(N). The H-atoms bonded to the O atom of the water molecule were found from difference Fourier map and their coordinates were refined independently with U_iso_(H) = 1.5 U_eq_(O). The H-atom bonded to the O atom of the ethanol molecule and the NH_2_ H atoms were refined freely.

### Crystal data

**Table:**

C_14_H_16_N_6_O_6_·C_2_H_6_O·H_2_O	*Z* = 4
*M**_r_* = 428.41	*F*(000) = 904
Monoclinic, *P*2_1_/*c*	*D*_x_ = 1.355 Mg m^−^^3^
Hall symbol: -P 2ybc	Cu *K*α radiation, λ = 1.54184 Å
*a* = 7.5810 (2) Å	θ = 3.6–75.7°
*b* = 12.1216 (3) Å	µ = 0.94 mm^−^^1^
*c* = 23.0379 (7) Å	*T* = 294 K
β = 97.254 (3)°	Block, colourless
*V* = 2100.11 (10) Å^3^	0.41 × 0.32 × 0.26 mm

### Data collection

**Table:**

Oxford Diffraction Xcalibur diffractometer	4317 independent reflections
Radiation source: fine-focus sealed tube	2927 reflections with *I* > 2σ(*I*)
graphite	*R*_int_ = 0.035
Detector resolution: 10.2576 pixels mm^-1^	θ_max_ = 75.9°, θ_min_ = 3.9°
ω scans	*h* = −8→9
Absorption correction: multi-scan (ABSPACK in CrysAlis PRO; Oxford Diffraction, 2009)	*k* = −14→15
*T*_min_ = 0.700, *T*_max_ = 1.000	*l* = −27→28
15863 measured reflections	

### Refinement

**Table:**

Refinement on *F*^2^	Secondary atom site location: difference Fourier map
Least-squares matrix: full	Hydrogen site location: inferred from neighbouring sites
*R*[*F*^2^ > 2σ(*F*^2^)] = 0.043	H atoms treated by a mixture of independent and constrained refinement
*wR*(*F*^2^) = 0.125	*w* = 1/[σ^2^(*F*_o_^2^) + (0.0775*P*)^2^] where *P* = (*F*_o_^2^ + 2*F*_c_^2^)/3
*S* = 0.98	(Δ/σ)_max_ = 0.002
4317 reflections	Δρ_max_ = 0.27 e Å^−^^3^
298 parameters	Δρ_min_ = −0.18 e Å^−^^3^
0 restraints	Extinction correction: *SHELXL97* (Sheldrick, 2008), Fc^*^=kFc[1+0.001xFc^2^λ^3^/sin(2θ)]^-1/4^
Primary atom site location: structure-invariant direct methods	Extinction coefficient: 0.0014 (3)

### Special details

**Table:**

Geometry. All e.s.d.'s (except the e.s.d. in the dihedral angle between two l.s. planes) are estimated using the full covariance matrix. The cell e.s.d.'s are taken into account individually in the estimation of e.s.d.'s in distances, angles and torsion angles; correlations between e.s.d.'s in cell parameters are only used when they are defined by crystal symmetry. An approximate (isotropic) treatment of cell e.s.d.'s is used for estimating e.s.d.'s involving l.s. planes.
Refinement. Refinement of *F*^2^ against ALL reflections. The weighted *R*-factor *wR* and goodness of fit *S* are based on *F*^2^, conventional *R*-factors *R* are based on *F*, with *F* set to zero for negative *F*^2^. The threshold expression of *F*^2^ > σ(*F*^2^) is used only for calculating *R*-factors(gt) *etc*. and is not relevant to the choice of reflections for refinement. *R*-factors based on *F*^2^ are statistically about twice as large as those based on *F*, and *R*- factors based on ALL data will be even larger.

### Fractional atomic coordinates and isotropic or equivalent isotropic displacement parameters (Å^2^)

**Table:**

	*x*	*y*	*z*	*U*_iso_*/*U*_eq_	
C1	0.2929 (2)	0.25039 (12)	0.51014 (7)	0.0377 (4)	
C2	0.3041 (2)	0.27386 (13)	0.56962 (7)	0.0400 (4)	
H2	0.2219	0.3212	0.5831	0.048*	
C3	0.4375 (2)	0.22701 (13)	0.60902 (7)	0.0427 (4)	
C4	0.5626 (2)	0.15889 (14)	0.58852 (8)	0.0501 (4)	
H4	0.6537	0.1288	0.6145	0.060*	
C5	0.5520 (2)	0.13560 (14)	0.52931 (9)	0.0526 (4)	
H5	0.6362	0.0897	0.5157	0.063*	
C6	0.4171 (2)	0.18025 (13)	0.49020 (8)	0.0457 (4)	
H6	0.4096	0.1633	0.4506	0.055*	
C7	0.1457 (2)	0.29742 (13)	0.46895 (7)	0.0385 (4)	
C8	0.1357 (2)	0.41831 (13)	0.45552 (7)	0.0412 (4)	
C9	0.2828 (3)	0.58940 (15)	0.47140 (10)	0.0582 (5)	
H9A	0.2586	0.6042	0.4302	0.087*	
H9B	0.3987	0.6170	0.4860	0.087*	
H9C	0.1951	0.6251	0.4915	0.087*	
C10	−0.1273 (2)	0.07098 (14)	0.42197 (7)	0.0455 (4)	
C11	0.4352 (2)	0.25050 (14)	0.67237 (7)	0.0475 (4)	
C12	0.5777 (3)	0.31779 (15)	0.70685 (8)	0.0553 (5)	
C13	0.8581 (3)	0.4016 (2)	0.70538 (13)	0.0876 (8)	
H13A	0.9592	0.3958	0.6843	0.131*	
H13B	0.8902	0.3768	0.7449	0.131*	
H13C	0.8199	0.4771	0.7056	0.131*	
C14	0.0394 (3)	0.12867 (16)	0.70835 (9)	0.0583 (5)	
N1	0.01612 (18)	0.24187 (11)	0.44138 (6)	0.0408 (3)	
N2	0.00539 (19)	0.13309 (10)	0.45248 (6)	0.0446 (3)	
H2A	0.0812	0.1026	0.4785	0.053*	
N3	−0.2322 (3)	0.12126 (15)	0.37973 (8)	0.0628 (5)	
N4	0.3108 (2)	0.22112 (12)	0.70208 (7)	0.0528 (4)	
N5	0.1754 (2)	0.15785 (13)	0.67639 (7)	0.0560 (4)	
H5A	0.1739	0.1361	0.6408	0.067*	
N6	0.0482 (3)	0.17095 (18)	0.76207 (9)	0.0713 (5)	
O1	0.27699 (16)	0.47111 (9)	0.48126 (6)	0.0489 (3)	
O2	0.01418 (18)	0.46321 (10)	0.42578 (6)	0.0585 (4)	
O3	−0.14134 (19)	−0.02689 (10)	0.43545 (6)	0.0583 (4)	
O4	0.7144 (2)	0.33357 (12)	0.67710 (7)	0.0670 (4)	
O5	0.5688 (2)	0.35432 (13)	0.75522 (6)	0.0767 (5)	
O6	−0.0770 (2)	0.06677 (13)	0.68590 (7)	0.0725 (4)	
C15	−0.3706 (4)	0.4190 (2)	0.32573 (11)	0.0836 (8)	
H15A	−0.4259	0.3992	0.2869	0.100*	
H15B	−0.3366	0.4961	0.3249	0.100*	
C16	−0.4991 (4)	0.4054 (3)	0.36663 (16)	0.1227 (12)	
H16A	−0.6073	0.4428	0.3520	0.184*	
H16B	−0.4523	0.4361	0.4038	0.184*	
H16C	−0.5229	0.3283	0.3712	0.184*	
O7	−0.2166 (2)	0.35452 (12)	0.33981 (7)	0.0649 (4)	
O1W	−0.2726 (4)	−0.13725 (18)	0.67387 (11)	0.1195 (8)	
H1WA	−0.2003	−0.0837	0.6802	0.179*	
H1WB	−0.3699	−0.1212	0.6866	0.179*	
H3A	−0.212 (4)	0.188 (3)	0.3697 (13)	0.106 (10)*	
H3B	−0.317 (3)	0.084 (2)	0.3617 (10)	0.073 (7)*	
H6A	0.132 (4)	0.212 (2)	0.7761 (12)	0.095 (10)*	
H6B	−0.046 (4)	0.158 (2)	0.7812 (11)	0.081 (8)*	
H7	−0.156 (4)	0.376 (2)	0.3730 (13)	0.111 (10)*	

### Atomic displacement parameters (Å^2^)

**Table:**

	*U*^11^	*U*^22^	*U*^33^	*U*^12^	*U*^13^	*U*^23^
C1	0.0379 (8)	0.0285 (7)	0.0444 (8)	−0.0087 (6)	−0.0032 (6)	0.0021 (6)
C2	0.0412 (9)	0.0310 (8)	0.0458 (9)	−0.0047 (6)	−0.0024 (7)	−0.0002 (6)
C3	0.0459 (9)	0.0318 (8)	0.0466 (9)	−0.0088 (7)	−0.0094 (7)	0.0010 (6)
C4	0.0473 (10)	0.0358 (8)	0.0623 (11)	−0.0004 (7)	−0.0120 (8)	0.0012 (8)
C5	0.0463 (10)	0.0410 (9)	0.0688 (12)	0.0020 (7)	0.0005 (9)	−0.0042 (8)
C6	0.0502 (10)	0.0364 (8)	0.0495 (9)	−0.0061 (7)	0.0019 (8)	−0.0041 (7)
C7	0.0408 (8)	0.0352 (8)	0.0385 (8)	−0.0059 (6)	0.0007 (7)	0.0027 (6)
C8	0.0417 (9)	0.0356 (8)	0.0455 (9)	−0.0049 (7)	0.0022 (7)	0.0032 (7)
C9	0.0573 (11)	0.0344 (9)	0.0817 (14)	−0.0099 (8)	0.0041 (10)	0.0042 (9)
C10	0.0529 (10)	0.0399 (9)	0.0402 (8)	−0.0106 (7)	−0.0078 (7)	0.0024 (7)
C11	0.0530 (10)	0.0392 (9)	0.0459 (9)	−0.0043 (8)	−0.0110 (8)	0.0042 (7)
C12	0.0653 (13)	0.0402 (9)	0.0524 (11)	−0.0038 (8)	−0.0231 (9)	0.0046 (8)
C13	0.0760 (16)	0.0672 (14)	0.1084 (19)	−0.0257 (12)	−0.0323 (14)	−0.0023 (14)
C14	0.0591 (12)	0.0493 (11)	0.0636 (12)	0.0003 (9)	−0.0030 (10)	0.0152 (9)
N1	0.0460 (8)	0.0343 (7)	0.0397 (7)	−0.0063 (6)	−0.0043 (6)	0.0042 (5)
N2	0.0496 (8)	0.0340 (7)	0.0452 (7)	−0.0086 (6)	−0.0131 (6)	0.0074 (6)
N3	0.0705 (11)	0.0482 (10)	0.0599 (10)	−0.0145 (8)	−0.0302 (8)	0.0064 (8)
N4	0.0620 (10)	0.0447 (8)	0.0470 (8)	−0.0047 (7)	−0.0112 (7)	0.0032 (6)
N5	0.0623 (10)	0.0561 (10)	0.0474 (8)	−0.0137 (8)	−0.0021 (7)	−0.0009 (7)
N6	0.0813 (15)	0.0677 (12)	0.0659 (12)	−0.0023 (11)	0.0131 (11)	0.0088 (10)
O1	0.0457 (7)	0.0326 (6)	0.0661 (8)	−0.0088 (5)	−0.0019 (6)	0.0034 (5)
O2	0.0556 (8)	0.0396 (7)	0.0740 (9)	−0.0047 (6)	−0.0157 (7)	0.0123 (6)
O3	0.0735 (9)	0.0405 (7)	0.0540 (7)	−0.0196 (6)	−0.0186 (6)	0.0065 (5)
O4	0.0638 (9)	0.0579 (8)	0.0729 (9)	−0.0211 (7)	−0.0159 (7)	−0.0047 (7)
O5	0.0873 (11)	0.0771 (10)	0.0574 (9)	−0.0112 (8)	−0.0233 (8)	−0.0126 (7)
O6	0.0637 (9)	0.0672 (9)	0.0839 (11)	−0.0132 (7)	−0.0017 (8)	0.0137 (8)
C15	0.0932 (19)	0.0754 (16)	0.0761 (15)	0.0252 (13)	−0.0131 (14)	0.0127 (12)
C16	0.095 (2)	0.151 (3)	0.125 (3)	0.047 (2)	0.026 (2)	0.030 (2)
O7	0.0647 (9)	0.0672 (9)	0.0588 (8)	0.0062 (7)	−0.0071 (7)	0.0032 (7)
O1W	0.154 (2)	0.1015 (16)	0.1034 (16)	−0.0471 (15)	0.0175 (16)	0.0006 (12)

### Geometric parameters (Å, °)

**Table:**

C1—C6	1.389 (2)	C12—O4	1.326 (3)
C1—C2	1.392 (2)	C13—O4	1.454 (2)
C1—C7	1.484 (2)	C13—H13A	0.9600
C2—C3	1.392 (2)	C13—H13B	0.9600
C2—H2	0.9300	C13—H13C	0.9600
C3—C4	1.385 (3)	C14—O6	1.222 (2)
C3—C11	1.489 (2)	C14—N6	1.333 (3)
C4—C5	1.385 (3)	C14—N5	1.387 (3)
C4—H4	0.9300	N1—N2	1.3476 (17)
C5—C6	1.385 (2)	N2—H2A	0.8600
C5—H5	0.9300	N3—H3A	0.86 (3)
C6—H6	0.9300	N3—H3B	0.85 (3)
C7—N1	1.290 (2)	N4—N5	1.356 (2)
C7—C8	1.498 (2)	N5—H5A	0.8600
C8—O2	1.206 (2)	N6—H6A	0.84 (3)
C8—O1	1.3224 (19)	N6—H6B	0.90 (3)
C9—O1	1.453 (2)	C15—O7	1.408 (3)
C9—H9A	0.9600	C15—C16	1.448 (4)
C9—H9B	0.9600	C15—H15A	0.9700
C9—H9C	0.9600	C15—H15B	0.9700
C10—O3	1.234 (2)	C16—H16A	0.9600
C10—N3	1.325 (2)	C16—H16B	0.9600
C10—N2	1.377 (2)	C16—H16C	0.9600
C11—N4	1.283 (2)	O7—H7	0.88 (3)
C11—C12	1.499 (2)	O1W—H1WA	0.8501
C12—O5	1.209 (2)	O1W—H1WB	0.8495
			
C6—C1—C2	119.41 (15)	O4—C13—H13A	109.5
C6—C1—C7	120.66 (15)	O4—C13—H13B	109.5
C2—C1—C7	119.90 (15)	H13A—C13—H13B	109.5
C1—C2—C3	120.53 (16)	O4—C13—H13C	109.5
C1—C2—H2	119.7	H13A—C13—H13C	109.5
C3—C2—H2	119.7	H13B—C13—H13C	109.5
C4—C3—C2	119.55 (16)	O6—C14—N6	125.3 (2)
C4—C3—C11	122.79 (15)	O6—C14—N5	118.5 (2)
C2—C3—C11	117.63 (16)	N6—C14—N5	116.2 (2)
C3—C4—C5	120.03 (16)	C7—N1—N2	118.53 (13)
C3—C4—H4	120.0	N1—N2—C10	119.73 (13)
C5—C4—H4	120.0	N1—N2—H2A	120.1
C6—C5—C4	120.47 (18)	C10—N2—H2A	120.1
C6—C5—H5	119.8	C10—N3—H3A	121 (2)
C4—C5—H5	119.8	C10—N3—H3B	117.5 (16)
C5—C6—C1	119.98 (17)	H3A—N3—H3B	121 (2)
C5—C6—H6	120.0	C11—N4—N5	119.39 (15)
C1—C6—H6	120.0	N4—N5—C14	119.03 (16)
N1—C7—C1	125.35 (14)	N4—N5—H5A	120.5
N1—C7—C8	113.16 (14)	C14—N5—H5A	120.5
C1—C7—C8	121.49 (13)	C14—N6—H6A	122 (2)
O2—C8—O1	123.61 (15)	C14—N6—H6B	115.9 (16)
O2—C8—C7	125.05 (15)	H6A—N6—H6B	122 (3)
O1—C8—C7	111.33 (14)	C8—O1—C9	116.52 (14)
O1—C9—H9A	109.5	C12—O4—C13	116.15 (18)
O1—C9—H9B	109.5	O7—C15—C16	113.2 (2)
H9A—C9—H9B	109.5	O7—C15—H15A	108.9
O1—C9—H9C	109.5	C16—C15—H15A	108.9
H9A—C9—H9C	109.5	O7—C15—H15B	108.9
H9B—C9—H9C	109.5	C16—C15—H15B	108.9
O3—C10—N3	124.35 (16)	H15A—C15—H15B	107.8
O3—C10—N2	118.54 (15)	C15—C16—H16A	109.5
N3—C10—N2	117.12 (15)	C15—C16—H16B	109.5
N4—C11—C3	124.74 (15)	H16A—C16—H16B	109.5
N4—C11—C12	113.41 (16)	C15—C16—H16C	109.5
C3—C11—C12	121.77 (17)	H16A—C16—H16C	109.5
O5—C12—O4	124.16 (18)	H16B—C16—H16C	109.5
O5—C12—C11	124.6 (2)	C15—O7—H7	111 (2)
O4—C12—C11	111.25 (17)	H1WA—O1W—H1WB	109.6

### Hydrogen-bond geometry (Å, °)

**Table:**

*D*—H···*A*	*D*—H	H···*A*	*D*···*A*	*D*—H···*A*
O1W—H1WA···O6	0.85	2.05	2.879 (3)	166
O1W—H1WB···O5^i^	0.85	2.16	2.941 (3)	152
N3—H3A···O7	0.86 (4)	2.13 (4)	2.980 (2)	169 (3)
N3—H3B···O5^ii^	0.85 (3)	2.61 (2)	3.082 (2)	116.7 (18)
N5—H5A···O3^iii^	0.86	2.19	3.010 (2)	160
N6—H6A···O1W^iv^	0.84 (3)	2.34 (3)	3.137 (3)	158 (3)
N6—H6B···O7^v^	0.90 (3)	1.99 (3)	2.871 (3)	166 (2)
O7—H7···O2	0.88 (3)	1.96 (3)	2.801 (2)	158 (3)
O7—H7···N1	0.88 (3)	2.52 (3)	3.0677 (19)	121 (2)
C4—H4···O6^vi^	0.93	2.57	3.493 (2)	173
C13—H13C···N6^vii^	0.96	2.62	3.405 (3)	139

Symmetry codes: (i) −*x*, *y*−1/2, −*z*+3/2; (ii) *x*−1, −*y*+1/2, *z*−1/2; (iii) −*x*, −*y*, −*z*+1; (iv) −*x*, *y*+1/2, −*z*+3/2; (v) *x*, −*y*+1/2, *z*+1/2; (vi) *x*+1, *y*, *z*; (vii) −*x*+1, *y*+1/2, −*z*+3/2.

## References

[bb1] Ismatov, D. N., Azizov, U. M., Leonteva, L. I., Zakirov, A. U. & Yuldashev, S. Z. (2001). *Pharm. Chem. J.* **35**, 418–420.

[bb2] Ismatov, D. N., Azizov, U. M., Nurullaeva, M. K. & Iskandarov, S. I. (1991). *Pharm. Chem. J.*, **25**, 498–502.

[bb3] Ismatov, D. N., Leonteva, L. I., Azizov, U. M., Karshiev, D. N. & Zakirov, U. B. (1998). *Pharm. Chem. J.*, **32**, 593–594.

[bb4] Oxford Diffraction (2009). *CrysAlis PRO* Oxford Diffraction Ltd, Yarnton, England.

[bb5] Sheldrick, G. M. (2008). *Acta Cryst.* A**64**, 112–122. 10.1107/S0108767307043930 18156677

